# Patient and public involvement in HIV research: a mapping review and development of an online evidence map

**DOI:** 10.1002/jia2.26385

**Published:** 2024-11-11

**Authors:** David Jackson‐Perry, Ellen Cart‐Richter, David Haerry, Lindrit Ahmeti, Annatina Bieri, Alexandra Calmy, Marie Ballif, Chloé Pasin, Julia Notter, Alain Amstutz, the Swiss HIV Cohort Study Young Researchers’ Group, and the Swiss HIV Cohort Study

**Affiliations:** ^1^ Infectious Diseases Service University Hospital Lausanne, University of Lausanne Lausanne Switzerland; ^2^ School of Social Sciences, Education and Social Work Queen's University Belfast UK; ^3^ Lausanne HIV‐Community Council University Hospital Lausanne, University of Lausanne Lausanne Switzerland; ^4^ President of the patient organization “Positive Council” Bern Switzerland; ^5^ CLEAR Methods Center, Division of Clinical Epidemiology, Department of Clinical Research University Hospital Basel, University of Basel Basel Switzerland; ^6^ Division of Infectious Diseases University Hospital Geneva Geneva Switzerland; ^7^ Department of Infectious Diseases, Inselspital Bern University Hospital, University of Bern Bern Switzerland; ^8^ Institute of Social and Preventive Medicine (ISPM) University of Bern Bern Switzerland; ^9^ Division of Infectious Diseases and Hospital Epidemiology University Hospital Zürich Zürich Switzerland; ^10^ Institute of Medical Virology University of Zürich Zürich Switzerland; ^11^ Collegium Helveticum Zürich Switzerland; ^12^ Division of Infectious Diseases, Infection Prevention and Travel Medicine Cantonal Hospital St. Gallen St. Gallen Switzerland; ^13^ Oslo Centre for Biostatistics and Epidemiology Oslo University Hospital, University of Oslo Oslo Norway; ^14^ Population Health Sciences, Bristol Medical School University of Bristol Bristol UK

**Keywords:** evidence map, good participatory practice, HIV/AIDS, mapping review, patient and public involvement, patient engagement

## Abstract

**Introduction:**

Increasing evidence indicates the benefits of patient and public involvement (PPI) in medical research, and PPI is increasingly expected by funders and publishers. We conducted a mapping review of studies reporting examples of PPI implementation in HIV research, and developed an online evidence map to guide HIV researchers.

**Methods:**

We systematically searched Medline and Embase up until 18 August 2024, including search terms with variations for PPI and HIV. We extracted information from identified studies in duplicate and analysed the data descriptively and qualitatively to describe types of PPI models and reported benefits, challenges, and mitigation strategies. This study was co‐initiated and co‐led by people living with HIV.

**Results:**

We identified 17 studies reporting PPI in HIV research between 1992 and August 2024. Most PPI examples informed prospective clinical studies, but also qualitative research, questionnaire development, research priority setting and surveys. Ten studies described the number and characteristics of PPI members involved. We observed four PPI models, from a model that solely engaged PPI members for a specific task to a model whereby PPI representatives were integrated into the study team with decision‐making authority. Benefits reported included wider dissemination of research results, better understanding of research material and results, and higher levels of trust and learning between researcher and communities. The most commonly reported challenges were the lack of specific resources for PPI, differing levels of knowledge and expertise, concern about HIV status disclosure, and lack of diversity of the PPI team. Uneven power dynamics, tensions, and differing expectations between stake‐holder groups were also frequently noted.

**Conclusions:**

This mapping review summarizes published examples of PPI in HIV research for various phases of research. There is a clear need to strengthen the reporting on PPI processes in HIV research, for example by following the Guidance for Reporting Involvement of Patients and the Public (GRIPP) 2 guidelines, and developing guidance on its hands‐on implementation. We embedded PPI from study inception onwards, which potentially pre‐empted some of the challenges reported in the reviewed examples. The resulting online evidence map is a starting point to guide researchers on integrating PPI into their own research.

## 1 INTRODUCTION

People with lived experience of medical conditions can provide valuable input into research endeavours to ensure that they are relevant and adapted to their needs. Patient and public involvement (PPI) is the process whereby patients and members of the public work alongside researchers, from the definition of research priorities, grant writing, study conceptualization, research tool development, results interpretation, through to dissemination [1, 2, 3]. Increasing evidence highlights how PPI can improve research in terms of patient‐relevant outcomes, project implementation, acceptability of the proposed intervention, and participant recruitment and engagement [4, 5]. Further, funders and publishers increasingly require PPI as an integral part of medical research and funding assessment boards. Historically, the field of HIV research has championed innovative PPI efforts, spurred largely by the activism of people living with HIV [6, 7]. However, despite increasing evidence and its history in HIV, PPI activities are rarely reported on [8]. This lack of practical guidance on PPI with concrete examples may lead to suboptimal use of PPI and increased risk of tokenism [9, 10, 11].

We conducted a mapping review of published PPI activities in HIV research to inform the creation of an online evidence map, intended as a continuously growing, hands‐on, and easy‐to‐search online database for researchers applying PPI in HIV research.

## 2 METHODS

### 2.1 Study design

Using a mapping review approach [12, 13], we systematically searched and reviewed publications reporting on PPI examples in HIV research. We registered our protocol on Open Science Framework [14]. Based on the reported examples of PPI implementation found during the review, we developed an online evidence map.

### 2.2 Patient and public involvement

This study was initiated, designed and conducted by an interdisciplinary team including people living with HIV as part of a larger project on PPI in the Swiss context. We report on our own PPI aim, methods and activities according to the Guidance for Reporting Involvement of Patients and the Public (GRIPP2) checklist [15] (Table 1).

**Table 1:** Description of the role of PPI in our own study, based on the Guidance for Reporting Involvement of Patients and the Public (GRIPP2) checklist [15]

**Aim of PPI in this study**	To co‐produce a research project on PPI in HIV research with people living with HIV as members of the research team To draw on the expertise of people living with HIV at all stages of the research project To render research results accessible to wider HIV communities
**Methods used for PPI in this study**	People living with HIV actively participated in all stages of the project: defining the initial research idea, defining the research questions and methods, acquiring funding, drafting the study protocol, interpreting the results, and writing the manuscript PPI and other contributors met bi‐weekly to discuss the study progress
**Result of PPI in this study**	PPI contributed to enriching the interpretation of the results PPI brought concrete experience concerning previous PPI activities in HIV research to the development of the online evidence map
**Discussion on the extent to which PPI influenced this study overall**	PPI members contributed at all stages of the project, which was unlikely to have been initiated otherwise PPI contributed research ideas and perspectives beyond the scope of this study that may lead to further research projects
**Critical perspective on the inclusion of PPI in this study**	PPI members sometimes lacked the necessary time to commit to research processes due to other professional activities. However, this is arguably the case in any research project including multiple partners Uneven levels of methodological expertise were apparent across study members including but not limited to PPI members PPI members in this study are not representative of populations affected by HIV in Switzerland (all were white, middle‐class, cis‐gender, and over 50, two were men, and one a woman).

Abbreviation: PPI, patient and public involvement.

### 2.3 Search strategy and data sources

We systematically searched studies published before 18 August 2024, without language or year restrictions, in Medline/PubMed and Embase, both via Ovid. Search terms included one of the following terms on PPI: (“patient participation”/ or “community based participatory research”/) or (“patient participation” or “patient and public involvement” or “public and patient involvement” or “patient involvement” or “community based participatory research” or “stakeholder engagement” or “stakeholder involvement” or “community involvement” or “good participatory practice” or “community and stakeholder participation” or “community working group” or “community advisory group” or “HIV advocate” or “HIV advocate”), combined with one of the following terms for HIV (“HIV”/ or “Acquired Immunodeficiency Syndrome”/ or “HIV infections”/ or (HIV or “Human Immunodeficiency Virus” or “Acquired Immunodeficiency Syndrome”).

### 2.4 Study screening, eligibility, data extraction, and analysis

We conducted screening and data extraction using Covidence (www.covidence.org). We included original articles that provided examples of PPI implementation in their HIV research projects. We excluded conference abstracts and examples of PPI in HIV care/service provision as our focus is on research. We screened abstracts and assessed full‐text eligibility in duplicate by two independent researchers. We then extracted data from each eligible study, in duplicate, including: the year and country where the study was conducted; the overall aim of the study; the target study population; the characteristics of PPI members; and reported challenges and mitigation strategies. We categorized the study design as prospective clinical study, qualitative research, survey, preclinical, or observational data analysis, and the project phases when PPI was involved into research project planning, research project implementation, closure (results interpretation), and post‐closure (results dissemination) phase.

We performed qualitative content analysis and used the Alberta Health Services’ engagement spectrum [16] as a framework to inductively define four categories of PPI models, and reported challenges and mitigation strategies. We highlighted quotes of relevant text passages, created labels that reflect the content, and collaboratively and iteratively modified and refined the final types of PPI models, benefits, challenges, and mitigation strategies.

We thereafter created the online evidence map collaboratively with all co‐authors as an iterative process over several meetings. To give concrete guidance to HIV researchers, we produced separate maps by type of research project (prospective clinical study, qualitative research, survey, preclinical, and observational data analysis).

### 2.5 Ethics statement

No ethical approval was required for this mapping review.

## 3 RESULTS

### 3.1 The review

We identified a total of 2239 records across all databases. After the removal of 1233 duplicates and 957 ineligible studies during the title/abstract screening process, we included 48 studies in the full‐text screening. We further excluded 31 studies for the following reasons: only conference abstract available (*n* = 15); the study examines PPI on a theoretical level without providing guidance (*n* = 10); not about HIV (*n* = 2); not about HIV research (only HIV service provision) (*n* = 4). The remaining 17 studies were extracted in detail [17, 18, 19, 20, 21, 22, 23, 24, 25, 26, 27, 28, 29, 30, 31, 32, 33].

### 3.2 Characteristics of included studies and PPI members

The included studies (Table 2) provide descriptions of PPI implementation examples used in their HIV research ranging from 1992 [19] to 2022, mostly in the last 10 years. Geographical scope included examples from southern Africa [17, 18, 21], North America [19, 20, 22, 23, 25, 26, 27, 31] and the United Kingdom [24, 28, 29, 30, 32, 33]. Most examples involved prospective clinical studies [17, 19, 20, 22, 24, 26, 27, 28, 30, 31, 32, 33], but also qualitative research [23], research instrument/questionnaire development [25, 26], research priority setting [21, 29], and surveys [19]. The included studies reported on PPI for research targeting people at risk for HIV acquisition (Latina women [22], sex workers [17], homelessness [30]), breastfeeding mothers living with HIV [18], men living with HIV in the military service [19], people living with HIV with cognitive impairment [29], and youth living with HIV [21, 23, 33]. In some studies, PPI was included in all phases of research, such as examples involving youth living with HIV in Canada [23] or South Africa [17], people at risk for HIV acquisition attending dental services [30], diverse communities of people living with HIV in the UK [28], or men who have sex with men and trans women in the UK [24]. Four studies provided specific PPI examples for the post‐closure phase [22, 25, 26, 33]. Ten studies [18, 19, 21, 23, 25, 27, 29, 30, 32, 33] described in detail how many PPI members were involved and their characteristics.

**Table 2:** Characteristics of the included studies and the reported PPI examples

Study ID	Country of the PPI example(s)	Year of the PPI example(s)	Example(s) for which type(s) of research	Example(s) for which phase(s) of research	Example(s) for which target study population	Characteristics of the involved PPI members	PPI model(s) reported^a^	Reported benefits	Reported challenges and mitigation strategies
Alford 2024 [29]	United Kingdom	2022	Research Priority Setting	Planning Phase	People living with HIV with cognitive impairment (dementia, HIV‐associated neurocognitive disorder)	Detailed description provided: Research Advisory Group: 15 lived experience, academic, healthcare and third sector professionals Two advisory focus groups: five people living with HIV with cognitive impairment (Male 4 [80%]; median age 59 [range 56–78]; White British 3 [60%], Black Caribbean 1 [20%], White other 1 [20%]; men who have sex with men 3 [60%], heterosexual 1 [20%], bi‐sexual 1 [20%], and three healthcare and third sector professionals (2 [66%] third sector professionals working at two local Brighton HIV charities and 1 [33%] HIV‐specialist clinical psychologist)	Model C: A few PPI representatives are part of study team, and a group of PPI representatives is consulted regularly	The research agenda is more PPI‐centred and not solely based on priority setting from provider perspective, thus understandable to the target study population and fostering mutual respect	*Challenges*: Representativeness (lack of diversity among PPI team), especially regarding gender
Baron 2018 [17]	South Africa	2011−2018	Prospective Clinical Study	All phases	People at risk for HIV acquisition (focus on sex workers and youth)	No description provided	A mix of PPI models reported (Models B−D)	Research team's socio‐cultural competency strengthened; PPI contributes to scientific and ethical integrity, accountability and transparency; Locally acceptable and understandable informed consent procedure and research material; Research conduct more PPI‐centred fostering trust and respect in the community; Research outcomes are disseminated more widely and are understandable and better applicable in the community; Post‐trial participant benefits maximized	*Challenges*: Adequate resources and funding for PPI activities; PPI as add‐on versus integral part; How to measure impact of PPI activity; Lack of documented evidence for PPI impact *Mitigation*: Early commitment to PPI activities (funding proposal stage); Adequate funding earmarked for PPI activities; Request progress of PPI activities in reports to the DMSB and other trial committees; Capture not only study‐specific recruitment and retention rates or number of people who attend PPI activities but also broader indicators (e.g. rumours in the community, research literacy levels, clinic accessibility); Create evidence‐based tools and guidance
Carey 1992 [19]	United States of America	1992	Prospective Clinical Study and Survey	Planning Phase and Implementation Phase	Men living with HIV in the military service	Detailed description provided: one protocol advisor, who is a person living with HIV with military experience and part of the military HIV programme; and the advisory group consisting of participants enrolled in the research study	Model A: A few PPI representatives as part of study team (with decision power; here: protocol advisor); Model C: A few PPI representatives are part of study team and a group of PPI representatives is consulted regularly	Locally acceptable and understandable informed consent procedure and research material; Research conduct more PPI‐centred fostering trust and respect in the community	No PPI‐related challenges reported
Corneli 2007 [18]	Malawi	2004	Prospective Clinical Study	Planning Phase	Breastfeeding mothers living with HIV	Detailed description provided: 40 mothers living with HIV, of infants less than 1‐year‐old; pregnant/breastfeeding women; from communities of the target study population	Model D: One‐off task‐focused PPI	Locally acceptable and understandable informed consent procedure and research material; Research conduct more PPI‐centred fostering trust and respect in the community	*Challenges*: Tensions between different stakeholder groups; Differing levels of understanding of PPI members about health research methods; Use of jargon; Time and resources; Representativeness (lack of diversity among PPI team) *Mitigation*: Cultural sensitivity (e.g. PPI conducted by people of same background/nationality and language); Clear explanation of health research methods including training material tailored to the different stakeholders involved; Independent facilitation (not the study principal investigator); Adequate funding earmarked for PPI activities
Day 2020 [20]	United States of America	2020	Prospective Clinical Study	Planning Phase	Members of the general public, 18 years of age or older	Vague description provided: Members of the general public, 18 years of age or older	Model D: One‐off task‐focused PPI	Locally acceptable and understandable informed consent procedure and research material	*Challenges*: General public may not understand what is feasible to implement in the clinical research project and thus provide unfeasible feedback; Communicating trial compensation amounts may create confusion among potentially future trial participants *Mitigation*: Concise and comprehensive training and dissemination material
Denison 2017 [21]	Southern Africa	2016	Research Priority Setting	Planning Phase	Youth living with HIV in southern Africa	Detailed description provided: four youth living with HIV (three women, one man) from southern Africa	Model D: One‐off task‐focused PPI	The research agenda is more PPI‐centred and not solely based on priority setting from provider perspective, thus understandable to the target study population and fostering mutual respect; Improved PPI collaboration during the entire subsequent research	*Challenges*: Specific financial and logistical challenges with youth living with HIV in southern Africa involved minors who had to travel to PPI meetings *Mitigation*: Engage similar older adolescents to accompany and support
Doughty 2024 [30]	United Kingdom	2022	Prospective Clinical Study	All phases	People at risk for HIV acquisition who attend dental services (focus on homelessness)	Detailed description provided: PPI contributors (among them one key contributor): three people who regularly attended the dentist who did not have a known diagnosis of HIV, three people who had lived experience of homelessness and had accessed homelessness‐specific dental services, and four people living with HIV. Within the group of people living with HIV were heterosexuals, men who have sex with men, women, people of White and Black African ethnicity	Model C: A few PPI representatives are part of study team and a group of PPI representatives is consulted regularly	Locally acceptable and understandable research material; Research outcomes are disseminated more widely and are understandable and better applicable in the community; Research team's socio‐cultural competency strengthened; Improvement of grant application	*Challenges*: PPI remained mostly collaborative and consultative but not co‐led; Time and resources; Ongoing power dynamic; Differing levels of understanding of PPI members about health research methods; How to measure impact of PPI activity *Mitigation*: Offer training and mentoring in PPI for research staff; Clear terms of references and memorandum of understanding (agreements)
Dubé 2023 [31]	United States of America	2016	Prospective Clinical Study	All phases	People living with HIV (focus on HIV cure)	Vague description provided: Three independent sets of partners: (1) an established community‐based organization providing health and social services to people living with HIV; (2) a cure‐directed community‐advisory board; (3) basic, clinical/biomedical and social scientists	Model C: A few PPI representatives are part of study team and a group of PPI representatives is consulted regularly	The research agenda is more PPI‐centred and not solely based on priority setting from provider perspective, thus understandable to the target study population and fostering mutual respect; Research team's socio‐cultural competency strengthened; Improved PPI collaboration during the entire subsequent research	*Challenges*: Time and resources; Tensions between different stakeholder groups; Lack of clarity about roles and decision‐making; Differing levels of understanding of PPI members about health research methods; Conflating community engagement with recruitment efforts for clinical trials and participation in research *Mitigation*: Early commitment to PPI activities (funding proposal stage); Adequate funding earmarked for PPI activities; Clear terms of references and memorandum of understanding (agreements); Review of enrolment totals with mandates to support the recruitment of specific study presented not allowed
Flaskerud 1999 [22]	United States of America	1999	Prospective Clinical Study	Planning Phase and Implementation Phase and Post‐Closure Phase	Latina women community at risk for HIV acquisition	Vague description provided: Members shared the ethnicity, language and culture of the other participants in the study	Model C: A few PPI representatives are part of study team and a group of PPI representatives is consulted regularly	Locally acceptable and understandable research material; Research outcomes are disseminated more widely and are understandable and better applicable in the community	No PPI‐related challenges reported
Flicker 2008 [23]	Canada	2002−2004	Qualitative Research (in‐depth interviews)	All phases	Youth living with HIV	Detailed description provided: 27 young people, identifying as being present or past members of at least one socially excluded community (e.g. gay, injection‐drug‐using or homeless communities)	Model C: A few PPI representatives are part of study team and a group of PPI representatives is consulted regularly	Research team's socio‐cultural competency strengthened; Locally acceptable and understandable informed consent procedure and research material; Activity itself fostering trust and mutual respect and learning between PPI and researchers; PPI offered different perspectives and better understanding of the data; More productive research output; Outcomes are disseminated more widely and are understandable and better applicable in the community	*Challenges*: Using PPI slows down the project pace (“more back and forth”); Increased workload; Added responsibility towards more stakeholders; Finding funding with appropriate balance between science and PPI; Ongoing power dynamic; Lack of clarity about roles and decision‐making; Risk of disclosure/stigma (impeded the dissemination work among others) *Mitigation*: Adequate funding earmarked for PPI activities; Clear terms of references and memorandum of understanding (agreements); Anticipate increased workload and longer study duration; Outsource/delegate to independent coordinators
Gafos 2020 [24]	United Kingdom	2015−2017	Prospective Clinical Study	All Phases	Men who have sex with men and transgender women	Vague description provided: Age and residence location	Model C: A few PPI representatives are part of study team and a group of PPI representatives is consulted regularly	Locally acceptable and understandable informed consent procedure and research material; Research conduct more PPI‐centred fostering trust and respect in the community; Research outcomes are disseminated more widely and are understandable and better applicable in the community	*Challenges*: PPI activity location (location familiar to some, but not others); Not enough action/communication after providing feedback; Time and resources; Differing levels of understanding of PPI members about health research methods; Risk of disclosure/stigma (impeded the dissemination work among others); Differing expectations between researchers and PPI members *Mitigation*: Choose “neutral” PPI activity location; Early commitment to PPI activities (funding proposal stage); Representation on advisory boards; Include people with PPI expertise; Adequate funding earmarked for PPI activities; Offer training and mentoring in PPI for research staff; Clear terms of references and memorandum of understanding (agreements)
Gobin 2024 [32]	United Kingdom	2022	Prospective Clinical Study	Planning phase	People at risk for HIV acquisition	Detailed description provided: The workshops included nine young people (six female), four gay, bisexual and other men who have sex with men and six from Black communities (all female)	Model B: The same group of PPI representatives is consulted regularly throughout the study	Locally acceptable and understandable informed consent procedure and research material	*Challenges*: Representativeness (lack of diversity among PPI team)
Lessard 2019 [25]	Canada	2015−2017	Research instrument/questionnaire development	Planning Phase and Post‐Closure Phase	People living with HIV in Quebec, Canada	Detailed description provided: 10 diverse members living with HIV in Quebec: man who has sex with men, man who has sex with women, person who inject drugs, queer, woman who has sex with men, African origin, White/European origin	Model B: The same group of PPI representatives is consulted regularly throughout the study	Research team's socio‐cultural competency strengthened; Locally acceptable and understandable research material; Activity itself fostering trust and mutual respect and learning between PPI and researchers	*Challenges*: Risk of disclosure/stigma (impeded the dissemination work among others); Differing expectations between researchers and PPI members; Professional background of PPI members sometimes ignored; Representativeness (lack of diversity among PPI team) *Mitigation*: Ensure anonymity (e.g. co‐authoring a publication under a collective label); Clear terms of references and memorandum of understanding (agreements); Explore alternative PPI methods to achieve a more inclusive PPI group (online, collaborate more closely with community‐based patient groups)
Lessard 2020 [26]	Canada	2018	Prospective Clinical Study and Research instrument/questionnaire development	Planning Phase and Post‐Closure Phase	People living with HIV in Quebec, Canada	Vague description provided: Probably most were Caucasian men, some with migration background and at least one female patient of African origin	Model C: A few PPI representatives are part of study team and a group of PPI representatives is consulted regularly (here: a “Patient Engagement agent”)	Not reported, only challenges and mitigation strategies	*Challenges*: Differing levels of understanding of PPI members about health research methods; PPI members diverted from initially assigned tasks; Time and resources; Risk of disclosure/stigma (impeded the dissemination work among others); Representativeness (lack of diversity among PPI team) *Mitigation*: Clear terms of references and memorandum of understanding (agreements); Allow for some flexibility in the agreement of PPI tasks; broaden the PPI activity (e.g. add an additional type of PPI model); Anticipate increased workload and longer study duration; Increasing emphasis on academic or professional experience and tailor involvement accordingly
Mott 2008 [27]	United States of America	2003	Prospective Clinical Study	Planning Phase and Implementation Phase	African American HIV serodifferent heterosexual couples	Detailed description provided: four sites totalled 62, with each site's Community Advisory Board having between 13 and 19 stakeholders: Black or African American or working in organizations that serve Black individuals	Model B: The same group of PPI representatives is consulted regularly throughout the study	Research team's socio‐cultural competency strengthened; locally acceptable and understandable informed consent procedure and research material; Research conduct more PPI‐centred fostering trust and respect in the community; Activity itself fostering trust and mutual respect and learning between PPI and researchers; Research outcomes are disseminated more widely and are understandable and better applicable in the community	*Challenges*: Differing levels of understanding of PPI members about health research methods; PPI members diverted from initially assigned tasks *Mitigation*: Clear terms of references and memorandum of understanding (agreements); Clear explanation of health research methods including training material tailored to the different stakeholders involved; Adequate funding earmarked for PPI activities; Offer training and mentoring in PPI for research staff
South 2016 [28]	United Kingdom and global	2003−2011	Prospective Clinical Study	All Phases	Diverse group of people living with HIV or at risk for HIV acquisition, in United Kingdom/global	Vague description provided: Members representing the target participant population	A mix of PPI models (Models A−D)	Locally acceptable and understandable informed consent procedure and research material; Research conduct more PPI‐centred fostering trust and respect in the community; Improved study recruitment; PPI offered different perspectives and better understanding of the data; Research outcomes are disseminated more widely and are understandable and better applicable in the community	No PPI‐related challenges reported
Sturgeon 2024 [33]	United Kingdom	2018	Prospective Clinical Study	Post‐Closure Phase	Youth living with HIV	Detailed description provided: 10 PPI contributors aged between 15 and 21 years (five female, five male), all taking antiretroviral treatment. Three were invited cohort participants and seven members of the Youth Trials Board at the Children's HIV Association	Model B: The same group of PPI representatives is consulted regularly throughout the study	Research outcomes are disseminated more widely and are understandable and better applicable in the community	*Challenges*: Ongoing power dynamic; Location where PPI activity was held was not ideal; Time and resources; Limited ownership of the final dissemination products *Mitigation*: Clear terms of references and memorandum of understanding (agreements); Offer training and mentoring in PPI for research staff; Consider appropriate PPI location

Abbreviation: PPI, patient and public involvement.

^a^ PPI models were classified deductively into four categories: Model A, where only one to three PPI representatives are part of the study team and have decision‐making capacity; Model B, whereby a group of PPI representatives is consulted regularly throughout the study but are not part of the study team and have no decision‐making capacity; Model C, whereby one to three PPI representatives are part of the study team with decision‐making capacity plus a group of PPI representatives is consulted regularly; Model D, whereby PPI representatives are only consulted for a one‐off task.

### 3.3 Reported PPI models

The following models were identified: Model A, where only one to three PPI representatives are part of the study team and have decision‐making capacity (2 examples [19, 28]); Model B, whereby a group of PPI representatives is consulted regularly throughout the study but are not part of the study team and have no decision‐making capacity (6 examples [17, 25, 27, 28, 32, 33]); Model C, whereby one to three PPI representatives are part of the study team with decision‐making capacity plus a group of PPI representatives is consulted regularly to increase the number and diversity of opinions (10 examples [17, 19, 22, 23, 24, 26, 28, 29, 30, 31]); Model D, whereby PPI representatives are only consulted for a one‐off task, for example input on the questionnaire or study protocol (5 examples [17, 18, 20, 21, 28]).

### 3.4 Reported benefits, challenges and mitigation strategies

The most frequently reported benefits were wider dissemination of research results, better understanding of consent forms, research materials, and results, higher levels of trust, respect, and learning between researcher and communities, better applicability of the findings in the community, and increased socio‐cultural competency of the research team. The challenges emerged around four key topics. First, limited resources, particularly time and funds for PPI activities, highlight the need for early PPI involvement for concrete planning of PPI and dedicated study staff for PPI activities. Second, differing levels of knowledge and expertise among PPI members and researchers was a recurring theme. This may be mitigated by offering training and defining clear terms and agreements. Third, risk of disclosure and stigma especially during publication and result dissemination, resulting in a need for publication strategies where individuals not wishing to be identified could still participate (e.g. collective co‐authorship and pseudonyms). Fourth, many studies noted the lack of demographic diversity among PPI members. Finally, uneven power dynamics, and tensions and differing expectations between stake‐holder groups were frequently noted. Mitigation strategies proposed were clear terms of reference and training for both researchers and PPI contributors.

The resulting online evidence map, based on the mapping review results, is available through the homepage of the Swiss HIV Cohort Study (SHCS) in an online format to continuously add further examples (http://www.shcs.ch/315‐patient‐and‐public‐involvement‐ppi).

## 4 DISCUSSION

This mapping review of PPI in HIV research yielded 17 studies reporting a wide range of examples of PPI implementation. Four common PPI models emerged. A model whereby PPI members have decision‐making capacity throughout the study (Models A and C) conceivably aligns more strongly with PPI principles compared to occasional involvement (Models B and D). Group‐based models (Models B and C) may encompass more diverse views. One‐off PPI models might be more feasible and similarly effective in addressing specific problems that arise during a trial, such as recruitment issues, but may be more susceptible to tokenism. A mix of different models may be resource‐intensive yet more effective, maximizing PPI's potential impact on a study. Given that the literature on theoretical aspects of PPI is extensive and PPI is increasingly encouraged by funders and publishers, we expected more published examples in HIV research. However, the limited number of studies found in this review rather represents underreporting of PPI examples than actual PPI use in HIV research.

We identified two other reviews about PPI in HIV research [34, 35]. First, a mixed‐methods review on PPI for questionnaire instrument development in HIV research and care [34]. It identified 41 studies assessing 39 instruments. In 79% of the studies, the involvement was restricted to consultation, comparable to our Model D. Second, a systematic review specifically assessing PPI for clinical trials found 108 eligible studies [35]. The review focused on stakeholders other than patients. Most often stakeholder involvement happened during the protocol development phase and study recruitment, only 13% informed the closure or post‐closure research phase.

Reflecting on our own PPI activity, we embedded PPI from study inception onwards and the study was formally co‐led by people living with HIV, which mitigated and potentially pre‐empted some of the challenges reported in the reviewed examples. This is perhaps particularly true concerning the uneven power dynamics and tensions and differing expectations between stake‐holder groups that were reported. Further, funders understood the importance of PPI: financial resources were, therefore, built into the initial protocol and funding application and not a challenge. While we encountered time constraints for meetings and research members having varying areas of expertise, this was not specific to PPI members. Risk of disclosure was not an issue for PPI members here, since two are members of the SHCS Scientific Board, and the third also lives openly with HIV. However, in terms of diversity, we faced similar challenges described by others in this review. Including women, racialized and other marginalized communities, such as substance users, is an ongoing difficulty in all areas of HIV research that we still seem to find challenging [11, 36]. While a few included studies in this review outlined in detail how PPI members were approached and selected, more guidance is needed in this aspect.

Our study has some shortcomings. First, our search was restricted to HIV research, and PPI examples only involving people living with HIV or people at risk for HIV acquisition, but not other stakeholders (the “Public” in PPI). Second, we excluded original studies that only mentioned PPI, for example, in a standard PPI statement, without giving further details. We acknowledge that this article does not give a holistic picture of all PPI activities in HIV research. Several international funders such as the U.S. National Institutes of Health and many clinical trial networks worldwide mandate PPI, with PPI often already deeply embedded at the protocol development stage. However, these efforts are rarely published as examples for others to learn from, and hence were not picked up in this review. Instead of requesting an unsystematic generic PPI statement to describe the PPI processes, journals and funders could adopt the GRIPP2 guidance. Third, while the examples found in this mapping review provide a good basis for the resulting online evidence map, none of the examples were from Switzerland and may thus compromise applicability. As a strength, this research project was co‐created and co‐led by people living with HIV, and involved a systematic search according to mapping review guidance and rigorous data extraction in duplicate.

## 5 CONCLUSIONS

In conclusion, this mapping review summarizes published examples of PPI in HIV research for various phases of research. There is a clear need to strengthen the reporting on PPI processes in HIV research, for example by following the GRIPP2 guidelines. Better hands‐on guidance for the implementation of PPI in HIV research is warranted. The resulting online evidence map is a starting point to guide future researchers on how to integrate and apply PPI in their own research.

## COMPETING INTERESTS

The authors report no competing interests.

## AUTHORS’ CONTRIBUTIONS

DJ‐P, DH, CP, JN and AA conceptualized the study and obtained the funding. LA, AB and AA conducted the search and data extraction, with input from DJ‐P, EC‐R, DH, CP, MB and JN. DJ‐P and AA drafted the first version of the manuscript. All named authors regularly reviewed the draft manuscript, provided input and approved the final version.

## FUNDING

This mapping review is part of a larger mixed methods study that aims to assess the attitudes and levels of knowledge of HIV research stakeholders in Switzerland on PPI and at providing concrete guidance on meaningful PPI for the SHCS and beyond. This overarching study is funded by the SHCS (project no #911) and Gilead Sciences. AA receives his salary from a postdoc.mobility grant of the Swiss National Science Foundation (#P500PM_221961).

## MEMBERS OF THE SWISS HIV COHORT STUDY YOUNG RESEARCHERS’ GROUP

Abela I, Amstutz A, Chammartin F, Ballif M, Damas J, Erba A, Kohns M, Leuzinger K, Marinosci A, Notter J, Pasin C, Salazar Vizcaya L, Surial B, Waldecker M.

## MEMBERS OF THE SWISS HIV COHORT STUDY

Abela I, Aebi‐Popp K, Anagnostopoulos A, Battegay M, Bernasconi E, Braun DL, Bucher HC, Calmy A, Cavassini M, Ciuffi A, Dollenmaier G, Egger M, Elzi L, Fehr J, Fellay J, Furrer H, Fux CA, Günthard HF (President of the SHCS), Hachfeld A, Haerry D (deputy of “Positive Council”), Hasse B, Hirsch HH, Hoffmann M, Hösli I, Huber M, Jackson‐Perry D, Kahlert CR (Chairman of the Mother & Child Substudy), Kaiser L, Keiser O, Klimkait T, Kouyos RD, Kovari H, Kusejko K (Head of Data Centre), Labhardt N, Leuzinger K, Martinez de Tejada B, Marzolini C, Metzner KJ, Müller N, Nemeth J, Nicca D, Notter J, Paioni P, Pantaleo G, Perreau M, Rauch A (Chairman of the Scientific Board), Salazar‐Vizcaya L, Schmid P, Speck R, Stöckle M (Chairman of the Clinical and Laboratory Committee), Tarr P, Trkola A, Wandeler G, Weisser M, Yerly S.

## Data Availability

All included studies and all generated data are presented in this manuscript.
